# Purinergic inhibitory regulation of esophageal smooth muscle is mediated by P2Y receptors and ATP-dependent potassium channels in rats

**DOI:** 10.1186/s12576-024-00916-5

**Published:** 2024-04-23

**Authors:** Takahiko Shiina, Yuji Suzuki, Kazuhiro Horii, Tomoya Sawamura, Natsufu Yuki, Yuuki Horii, Yasutake Shimizu

**Affiliations:** 1 Department of Basic Veterinary Science, Laboratory of Physiology, Joint Graduate School of Veterinary Sciences, Gifu University, 1-1 Yanagido, Gifu, 501-1193 Japan; 2 Department of Basic Veterinary Science, Laboratory of Physiology, Joint Department of Veterinary Medicine, Faculty of Applied Biological Sciences, Gifu University, 1-1 Yanagido, Gifu, 501-1193 Japan; 3 Division of Biological Principles, Department of Physiology, Graduate School of Medicine, Gifu University, 1-1 Yanagido, Gifu, 501-1193 Japan; 4 Institute for Glyco-Core Research (iGCORE), Gifu University, 1-1 Yanagido, Gifu, 501-1193 Japan; 5 Division of Animal Medical Science, Center for One Medicine Innovative Translational Research (COMIT), Gifu University Institute for Advanced Study, 1-1 Yanagido, Gifu, 501-1193 Japan

**Keywords:** ATP-dependent potassium channel, Esophagus, P2 receptor, Purine, Smooth muscle

## Abstract

Purines such as ATP are regulatory transmitters in motility of the gastrointestinal tract. The aims of this study were to propose functional roles of purinergic regulation of esophageal motility. An isolated segment of the rat esophagus was placed in an organ bath, and mechanical responses were recorded using a force transducer. Exogenous application of ATP (10–100 μM) evoked relaxation of the esophageal smooth muscle in a longitudinal direction under the condition of carbachol (1 μM) -induced precontraction. Pretreatment with a non-selective P2 receptor antagonist, suramin (500 μM), and a P2Y receptor antagonist, cibacron blue F3GA (200 μM), inhibited the ATP (100 μM) -induced relaxation, but a P2X receptor antagonist, pyridoxal phosphate-6-azophenyl-2,4-disulfonic acid (50 μM), did not affect it. A blocker of ATP-dependent potassium channels (K_ATP_ channels), glibenclamide (200 μM), inhibited the ATP-induced relaxation and application of an opener of K_ATP_ channels, nicorandil (50 μM), produced relaxation. The findings suggest that ATP is involved in inhibitory regulation of the longitudinal smooth muscle in the muscularis mucosae of the rat esophagus via activation of P2Y receptors and then opening of K_ATP_ channels.

## Background

In mammalian esophagi, the muscularis propria contains not only smooth muscle fibers but also striated muscle fibers [1–3]. The composition of muscle cells in the muscularis propria is dependent on species [2–5]. In addition, there are longitudinal smooth muscle fibers in the esophageal muscularis mucosae, which are involved in esophageal shortening [7, 8]. The motor functions of the esophagus are controlled by extrinsic vagal neurons [9, 10] and by intrinsic myenteric neurons releasing various neurotransmitters such as acetylcholine, tachykinins, nitric oxide and galanin [2, 3, 10–15].

It is well known that gastrointestinal motility is regulated by various transmitters including acetylcholine, amines, lipid mediators, gas transmitters, peptides and purines such as adenosine, adenosine diphosphate (ADP) and adenosine triphosphate (ATP) [16–20]. Purinoceptors are classified into two families: receptors for adenosine (P1 receptors) and receptors for ATP and ADP (P2 receptors) [16, 17, 21, 22]. P2 receptors are separated into two groups based on their transduction mechanism. P2X receptors are ligand-gated ion channels and P2Y receptors are G protein-coupled receptors (GPCRs) [16, 17, 21, 22]. To date, seven P2X receptor subtypes (P2X1-7) and eight P2Y receptor subtypes (P2Y1, 2, 4, 6, 11–14) have been identified [23]. P2 receptors are expressed in the gastrointestinal tract including the esophagus [17, 23–26]. Previous studies demonstrated that ATP causes inhibitory responses of smooth muscles in the jejunum, ileum, caecum, and colon and suggested that ATP may be an inhibitory neurotransmitter in the gastrointestinal tract [17, 23, 24].

In the esophagus, purinoceptors have been detected histologically [25, 26] and pharmacologically [27]. In addition, it has been shown that ATP is released from esophageal epithelial keratinocytes in response to transient receptor potential vanilloid (TRPV) 4 channel activation [28]. However, the precise mechanism of purinergic regulation of esophageal motility is still unclear. Therefore, the aims of the present study were to determine the characteristics of mechanical responses induced by ATP in the rat esophagus and then propose functional roles of purinergic regulation of esophageal motility.

## Methods

### Animals

Male Wistar rats (*Rattus norvegicus*, 8–12 weeks of age, 200–250 *g* in weight) were obtained from Japan SLC (Shizuoka, Japan). They were maintained in plastic cages at 24 ± 2 ℃ with a 12:12-h light–dark cycle (light on at 7:00–19:00) and given free access to laboratory chow and water. The experiments were approved by the Gifu University Animal Care and Use Committee and were conducted in accordance with the committee guidelines on animal care and use (permission numbers: 14105, 15098, 17005, H30-183, 2019-239, 2020-252, 2021-255, 20220062).

### Esophageal tissue preparations

Animals were anesthetized with isoflurane and were exsanguinated via axillary arteries. A 1-cm-long segment from the middle thoracic part of the esophagus was dissected out. The segment of the esophagus was immediately immersed in Krebs’ solution (see below) at room temperature, and the intraluminal contents of the excised segment were flushed using a small cannula containing Krebs’ solution.

### Recording of mechanical activity in esophageal segments

For recording contractile responses in the longitudinal direction, the whole segment was mounted in a Magnus’s tube (10 mL in capacity) filled with Krebs’ solution (pH 7.4). One end of the esophageal segment was tied to the Magnus’s tube and the other end was secured with a silk thread to an isometric force transducer (T7-8-240, Orientec, Tokyo, Japan). The Krebs’ solution was continuously bubbled with a 95% O_2_ + 5% CO_2_ gas mixture and maintained at 37 ℃. Mechanical responses were filtered and amplified by an amplifier (NEC, AS1202, Tokyo, Japan) and recorded using a PowerLab system (AD Instruments, Bella Vista, NSW, Australia). An initial resting tension of 1.0 *g* was applied to the preparations, which were subsequently allowed to equilibrate for at least 30 min.

### Solutions and drugs

During experiments, tissues were maintained in Krebs’ solution consisting of (mM): NaCl 118.4, KCl 4.7, CaCl_2_ 2.5, MgSO_4_ 1.2, KH_2_PO_4_ 1.2, NaHCO_3_ 25 and glucose 11.7. ATP, tetraethylammonium chloride (TEA), tetrodotoxin, atropine sulfate monohydrate and carbachol were obtained from FUJIFILM-Wako (Osaka, Japan). D-tubocurarine, suramin, methoctramine hydrate, 4-diphenylacetoxy-N-methyl-piperidine methiodide (4-DAMP), pyridoxal phosphate-6-azophenyl-2,4-disulfonic acid **(**PPADS), cibacron blue F3GA (CBF3GA; synonym reactive blue 2) and 4-aminopyridine (4-AP) were obtained from Sigma-Aldrich (St Louis, MO, USA). Glibenclamide and nicorandil were obtained from Tokyo Chemical Industry (Tokyo, Japan). GSK1016790A was obtained from Cayman Chemical (Ann Arbor, MI, USA). Apamin was obtained from Peptide Institute (Osaka, Japan). Tetrodotoxin was dissolved in citrate solution. Glibenclamide, nicorandil and GSK1016790A were dissolved in DMSO. Other drugs were dissolved in distilled water. The highest concentration of vehicles (0.1%) for the drugs alone had no effect on the basal tone and contractile responses at the concentrations used. The concentrations of drugs given were final concentrations in the bath solution.

### RNA isolation and reverse transcription-polymerase chain reaction (RT-PCR)

The expression of P2 receptor gene and K_ATP_ channel gene mRNAs was assessed by RT-PCR. Total cellular RNA was extracted from tissue homogenates of the rat esophageal mucosa using TRI Reagent (Molecular Research Center, Cincinnati, OH, USA). First-strand cDNA was synthesized from 3 µg of total RNA by using SuperScript III Reverse Transcriptase (Thermo Fisher Scientific, Waltham, MA, USA) and Random primers (Thermo Fisher Scientific). The absence of PCR-amplified DNA fragments in the samples without reverse transcription indicated the isolation of RNA free from genomic DNA contamination. PCR was performed with Platinum Taq DNA Polymerase High Fidelity (Thermo Fisher Scientific). The primer sets are shown in Table 1. All primers were purchased from Thermo Fisher Scientific. Amplifications were performed by 35 cycles. The reaction products were electrophoresed on 1.5% agarose gels and stained with ethidium bromide (0.8 µg/mL). The gels were imaged with a UV transiluminator (UVP Laboratory Products, Upland, CA, USA) and photographed.

**Table 1:** List of primers for RT-PCR

Gene	Sequence (5ʹ- 3ʹ)	Predicted size (bp)
P2X1	Forward GCTGACTATGTCTTCCCAGC	454
	Reverse GACTCTCGCACCACATAGC	
P2X2	Forward GAATCAGAGTGCAACCCCAA	357
	Reverse TCACAGGCCATCTACTTGAG	
P2X3	Forward TGGCGTTCTGGGTATTAAGATCGG	440
	Reverse CAGTGGCCTGGTCACTGGCGA	
P2X4	Forward GAGGCATCATGGGTATCCAGATCAAG	447
	Reverse GAGCGGGGTGGAAATGTAACTTTAG	
P2Y1	Forward CCTGCGAAGTTATTTCATCTA	318
	Reverse GTTGAGACTTGCTAGACCTCT	
P2Y2	Forward CTGCCAGGCACCCGTGCTCTACTT	339
	Reverse CTGAGGTCAAGTGATCGGAAGGAG	
P2Y4	Forward CACCGATACCTGGGTATCTGCCAC	377
	Reverse CAGACAGCAAAGACAGTCAGCACC	
P2Y6	Forward GACCTTGCCTGCCGCCTGGTA	481
	Reverse TACCACGACAGCCATACGGGCCGC	
Kir6.1	Forward AAAGGAAGATGCTGGCCAGGAA	339
	Reverse CCGTGATGCCTTTCTCCATGTA	
Kir6.2	Forward CGCATGGTGACAGAGGAATG	297
	Reverse GTGGAGAGGCACAACTTCGC	
SUR1	Forward TGGGGAACGGGGCATCAACT	388
	Reverse TGGCTCTGGGGCTTTTCTC	
SUR2A	Forward TTGTTCGAAAGAGCAGCATAC	155
	Reverse GCCCGCATCCATAATAGAGG	
SUR2B	Forward TTGTTCGAAAGAGCAGCATAC	144
	Reverse GAATGGTGTGAACCCGATGAG	

### Data presentation and statistical analysis

Data are presented as means ± standard error of the mean (SE), and *n* indicates the number of separate preparations. The values of relaxation responses are maximum amplitudes of relaxations induced by application of ATP that are normalized as percentages of carbachol (1 µM)-induced contractions. The significance of differences between mean values was determined by the paired t-test for comparison of two groups. A *P* value less than 0.05 denotes the presence of a statistically significant difference.

## Results

### Effects of ATP on mechanical activity of rat esophageal segments

**Fig. 1:**
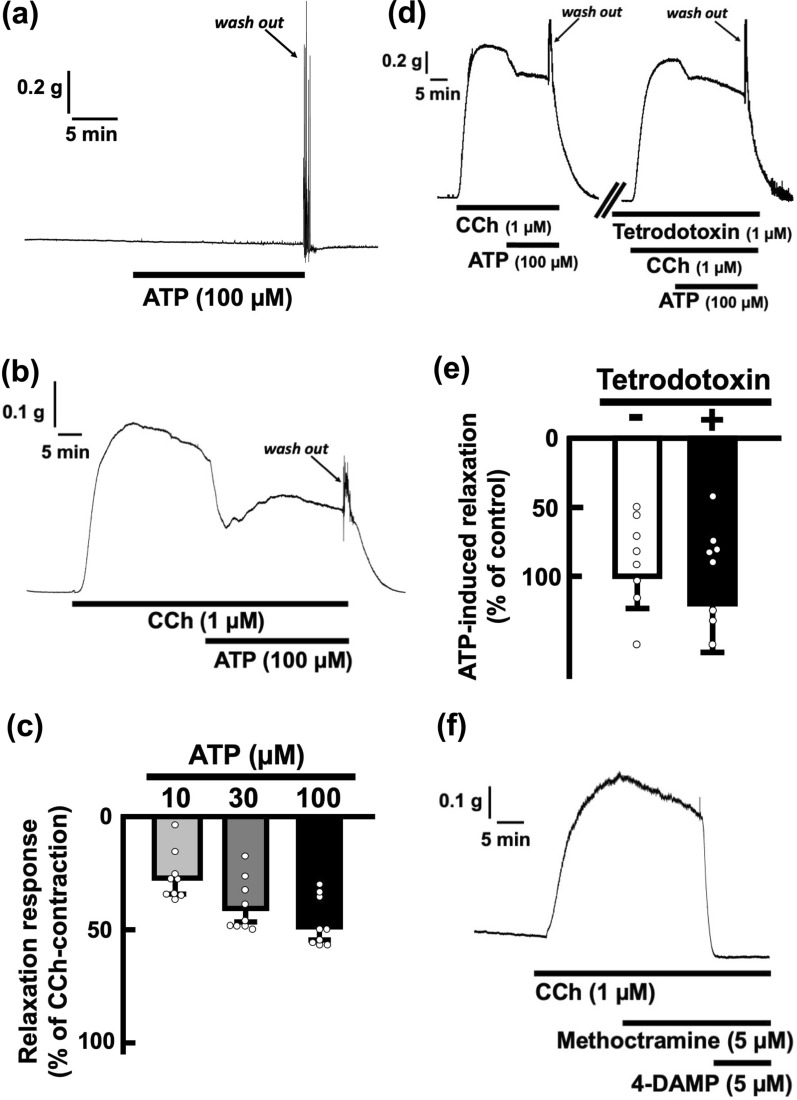
ATP-evoked relaxations in the rat esophagus. **a** A representative tracing demonstrating the effect of ATP on basal tone of the rat esophagus in longitudinal direction is shown. ATP was added to the organ bath (100 µM). **b** A representative tracing demonstrating the effect of ATP on longitudinal tension of the rat esophagus is shown. Administration of carbachol (CCh; 1 µM) induced contraction, and ATP was added to the organ bath (100 µM). **c** Dose-dependency of the relaxation response evoked by ATP in the rat esophagus is summarized (n = 9). The values of relaxation responses are normalized as percentages of CCh (1 µM)-induced contractions. **d** Representative tracings demonstrating the relaxation induced by ATP (100 µM) of the rat esophagus in the absence or presence of tetrodotoxin (1 µM) are shown. **e** Summary graphs of relaxation evoked by ATP (100 µM) in the absence or presence of tetrodotoxin are shown (n = 8). The values of relaxation responses are normalized as percentages of the control relaxation responses induced by ATP (100 µM) in the absence of tetrodotoxin. Each bar represents the mean of data ± standard error of the mean (SE). Dots show individual data. **f** A representative tracing demonstrating the effect of muscarinic acetylcholine receptor antagonists, methoctramine (5 µM), a selective M2 muscarinic receptor antagonist, and 4-DAMP (5 µM), a selective M3 muscarinic receptor antagonist, on carbachol-induced contractile response of the rat esophagus is shown. CCh, carbachol. 4-DAMP, 4-diphenylacetoxy-N-methyl-piperidine methiodide

Exogenous application of ATP (100 µM) did not affect basal tension of the rat esophageal segments (Fig. 1a). Application of carbachol (1 µM) induced a sustained contraction and then gradual relaxation occurred after the achievement of max contraction (Fig. 1b). The rat esophageal segment was precontracted with carbachol (1 µM), and then ATP (100 µM) was applied in the bath. Under this condition, ATP produced a relaxation (Fig. 1b). In some preparations, transient relaxation response was followed by contractile response or recovery of original tone. Washing out induced transient contractile response. After washing out, the tension returned to the basal level (Fig. 1b). The relaxation activity increased in a concentration-dependent manner (Fig. 1c). On the other hand, tetrodotoxin (1 µM), a blocker of voltage-dependent sodium channels on neurons and striated muscle, did not affect the carbachol-induced contraction and the ATP-induced relaxation of the rat esophagus (Fig. 1d, e). The carbachol-induced contraction was inhibited by atropine (5 µM), a blocker of muscarinic acetylcholine receptors on smooth muscle cells, but not by d- tubocurarine (5 µM) (data not shown). In addition, the carbachol-induced contraction was inhibited by 4-DAMP (5 µM), a selective M3 muscarinic receptor antagonist, but not by methoctramine (5 µM), a selective M2 muscarinic receptor antagonist (Fig. 1f).

### Effects of antagonists for purinoceptors on ATP-evoked relaxations in rat esophageal segments

**Fig. 2:**
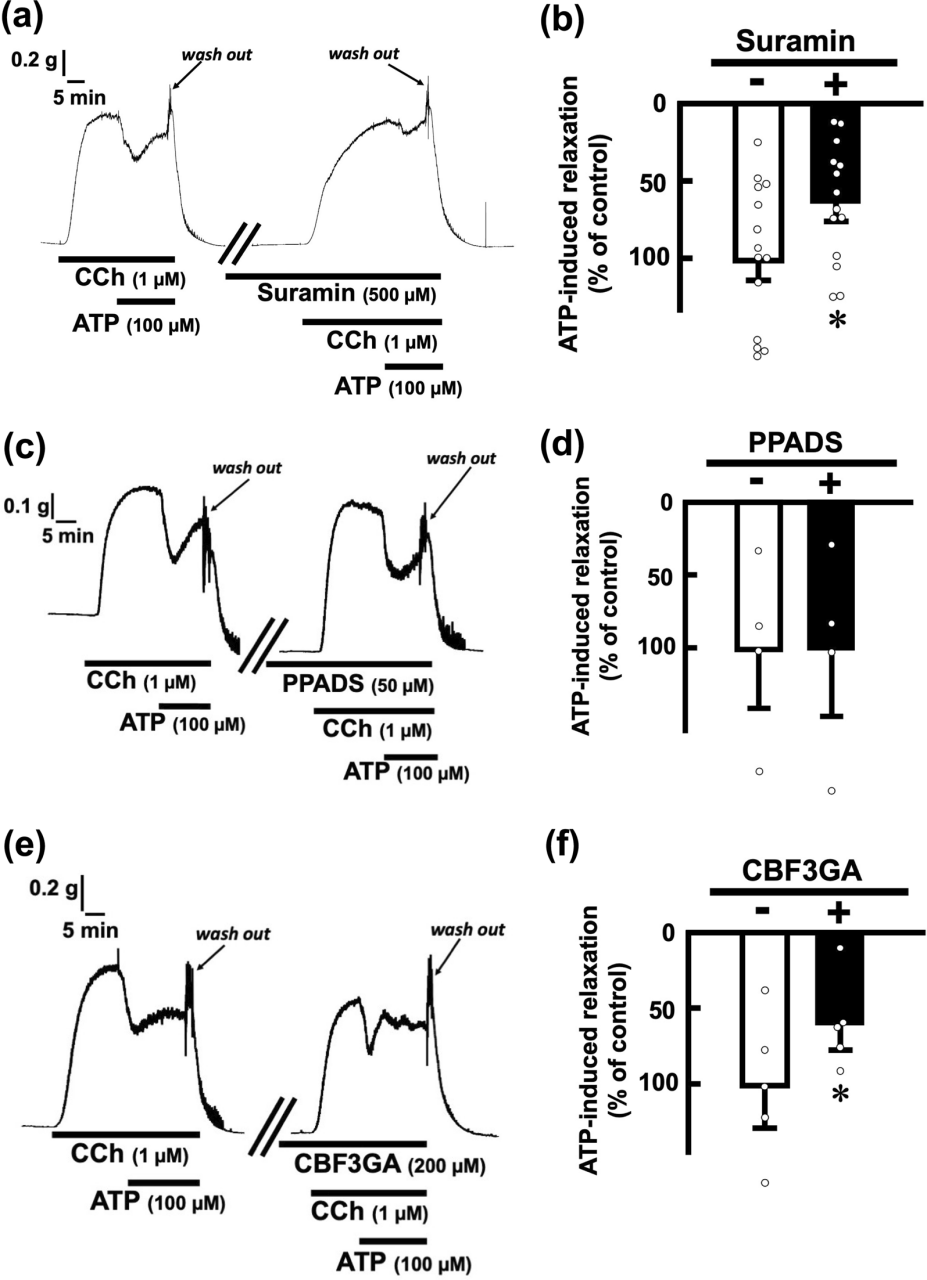
Effects of antagonists for P2 receptors on ATP-evoked relaxation in the rat esophagus. Representative tracings demonstrating the relaxation effect of ATP (100 µM) on longitudinal tension of the rat esophagus in the absence or presence of suramin (500 µM), a non-selective antagonist for P2 receptors (**a**), PPADS (50 µM), a selective antagonist for P2X receptors (**c**), and CBF3GA (200 µM), a selective antagonist for P2Y receptors (**e**), are shown. The inhibitory effects of suramin (500 µM) (**b**; n = 14), PPADS (50 µM) (**d**; n = 4), and CBF3GA (200 µM) (**f**; n = 5) on ATP (100 µM)-evoked relaxation in the rat esophagus are summarized. The values of relaxation responses are normalized as percentages of the control relaxation responses induced by ATP (100 µM) in the absence of indicated drugs. Each bar represents the mean of data ± SE. Dots show individual data. *P < 0.05, compared to the control. *CCh* carbachol, *PPADS* pyridoxal phosphate-6-azophenyl-2,4-disulfonic acid, *CBF3GA* cibacron blue F3GA

To determine whether ATP-evoked relaxations are mediated via purinoceptors, we examined the effects of antagonists for purinoceptors. Pretreatment with suramin (500 µM), a non-selective P2 receptor antagonist, blocked ATP (100 µM)-induced relaxations in rat esophageal segments (Fig. 2a, b). Next, we tested a selective antagonist for P2X receptors and a selective antagonist for P2Y receptors. Pretreatment with PPADS (50 µM), a P2X receptor antagonist, did not affect ATP-evoked relaxations (Fig. 2c, d). On the other hand, pretreatment with CBF3GA (200 µM), a P2Y receptor antagonist, inhibited the ATP-evoked relaxations (Fig. 2e, f).

### Involvement of potassium channels in ATP-evoked relaxations in rat esophageal segments

**Fig. 3:**
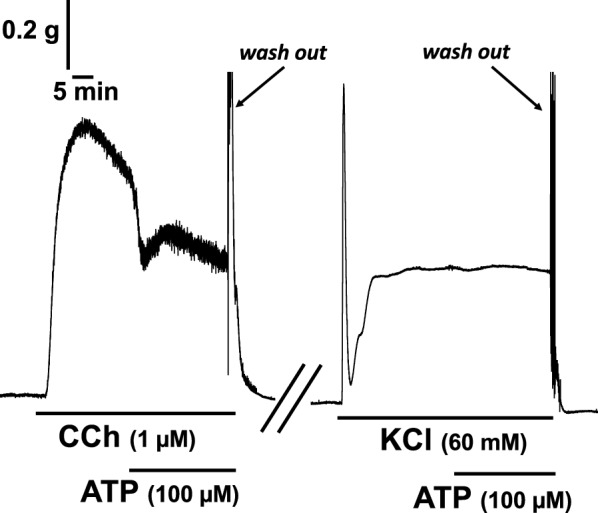
Effect of exogenous ATP on high concentration of potassium-evoked contractions in the rat esophagus. Representative tracings demonstrating the effect of ATP on longitudinal tension of the rat esophagus are shown (n = 3). Administration of carbachol (CCh; 1 µM) or KCl (60 mM) induced contraction, and ATP was added to the organ bath (100 µM). *CCh* carbachol

**Fig. 4:**
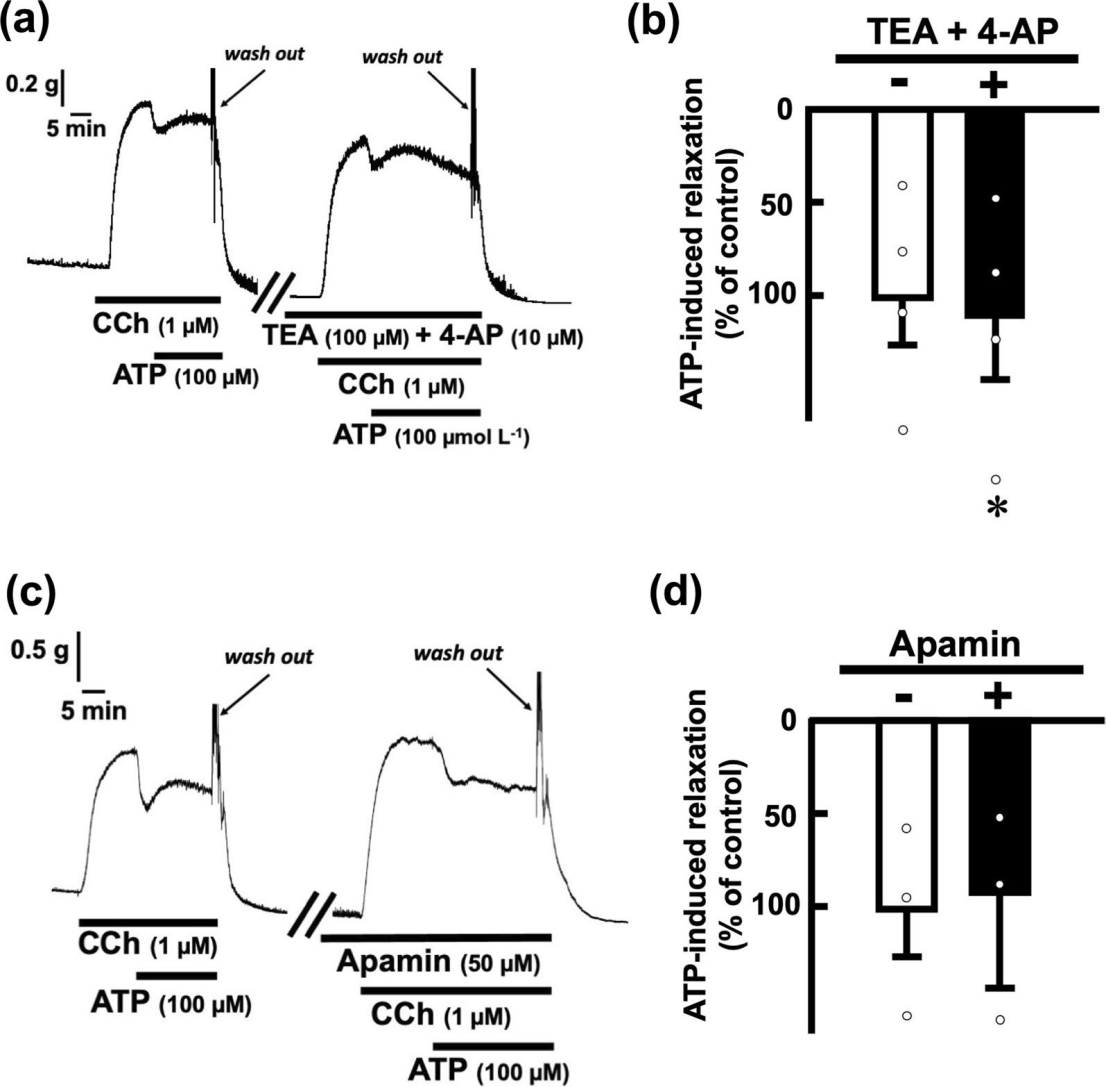
Effects of potassium channel closers on ATP-evoked relaxation in the rat esophagus. Representative tracings demonstrating the relaxation effect of ATP (100 µM) on longitudinal tension of the rat esophagus in the absence or presence of TEA (100 µM) and 4-AP (10 µM), voltage-dependent potassium channel closers (**a**), and apamin (50 µM) (**c**) are shown. The inhibitory effects of TEA (100 µM) and 4-AP (10 µM) (**b**; n = 4) and apamin (50 µM) (**d**; n = 3) on ATP (100 µM)-evoked relaxation in the rat esophagus are summarized. The values of relaxation responses are normalized as percentages of the control relaxation responses induced by ATP (100 µM) in the absence of indicated drugs. Each bar represents the mean of data ± SE. Dots show individual data. *P < 0.05, compared to the control. *CCh* carbachol, *TEA* tetraethylammonium chloride, *4-AP* 4-aminopyridine

**Fig. 5:**
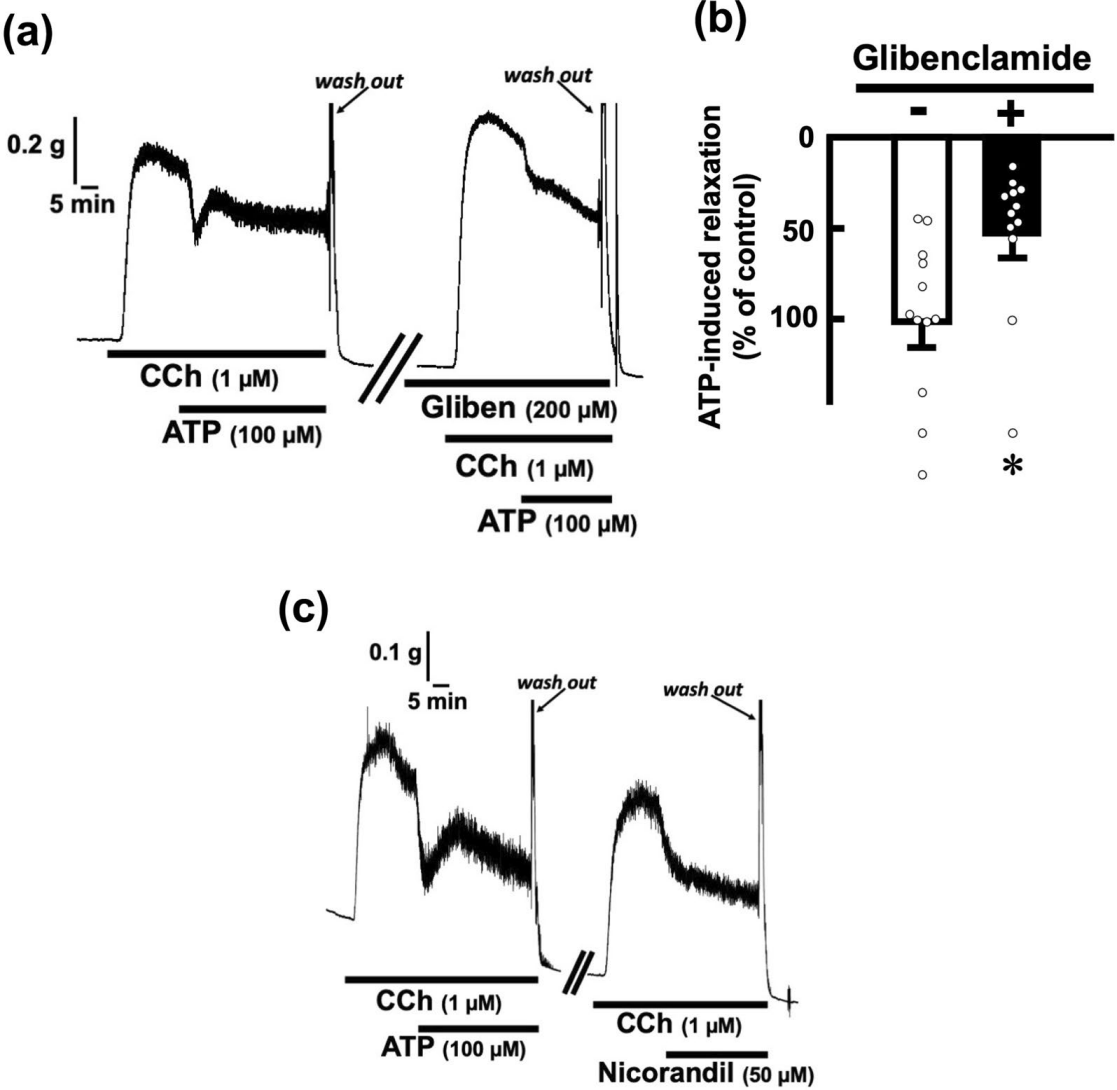
Effects of a closer of K_ATP_ channels on ATP-evoked relaxation and an opener of K_ATP_ channels on mechanical response in the rat esophagus. (**a**) Representative tracings demonstrating the relaxation effect of ATP (100 µM) on longitudinal tension of the rat esophagus in the absence or presence of glibenclamide (Gliben, 200 µM), a closer of K_ATP_ channels, are shown. (**b**) The inhibitory effects of glibenclamide (200 µM) (n = 12) on ATP (100 µM)-evoked relaxation in the rat esophagus are summarized. The values of relaxation responses are normalized as percentages of the control relaxation responses induced by ATP (100 µM) in the absence of indicated drugs. Each bar represents the mean of data ± SE. Dots show individual data. *P < 0.05, compared to the control. (**c**) Representative tracings demonstrating the relaxation responses induced by ATP (100 µM) and nicorandil (50 µM), an opener of K_ATP_ channels, are shown (n = 4). *CCh* carbachol, *Gliben* glibenclamide

Considering that P2Y receptors are GPCRs, which are linked to potassium channels, we examined whether potassium channels are involved in ATP-evoked relaxations in the rat esophagus. After application of KCl (60 mM) to induce contraction, ATP (100 µM) was applied in the bath. Under this condition, ATP did not induce relaxation (Fig. 3). To determine what potassium channels are involved in ATP-evoked relaxations in the rat esophagus, closers and openers of the channels were used. Even pretreatment with TEA (100 µM) and 4-AP (10 µM), voltage-dependent potassium channel closers, did not inhibit but rather enhanced the relaxation effect of ATP (Fig. 4a, b). Apamin, a calcium-dependent potassium channel closer, also did not affect ATP-induced relaxation (Fig. 4c, d). On the other hand, pretreatment with glibenclamide (200 µM), a closer of ATP-dependent potassium channels (K_ATP_ channels), blocked ATP (100 µM)-induced relaxations in rat esophageal segments (Fig. 5a, b). Application of nicorandil (50 µM), an opener of the channels, induced relaxation of the rat esophagus under the condition of precontraction with carbachol (1 µM) (Fig. 5c).

### Molecular identification of P2 receptors and KATP channels in the rat esophagus

**Fig. 6:**
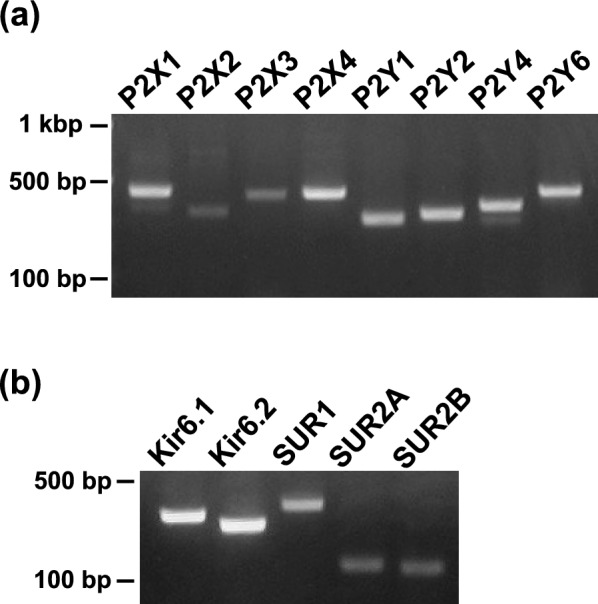
Expression of subtypes of P2 receptors (**A**) and subunits of K_ATP_ channels (**B**) in the rat esophageal mucosa determined by RT-PCR. Amplified products of mRNA of P2X1, P2X2, P2X3, P2X4, P2Y1, P2Y2, P2Y4, P2Y6, Kir6.1, Kir6.2, SUR1, SUR2A and SUR2B were detected in appropriate sizes (n = 3)

We then examined the expression of subtypes of P2 receptors and subunits of K_ATP_ channels in the rat esophageal mucosa by using RT-PCR. Amplified products of mRNA of several subtypes of P2X and P2Y receptors were observed in appropriate sizes (Fig. 6a). Subunits of K_ATP_ channels, Kir6.1, Kir6.2, SUR1, SUR2A and SUR2B, were also detected in appropriate sizes (Fig. 6b).

### Effects of a TRPV4 opener on mechanical activity of rat esophageal segments

**Fig. 7:**
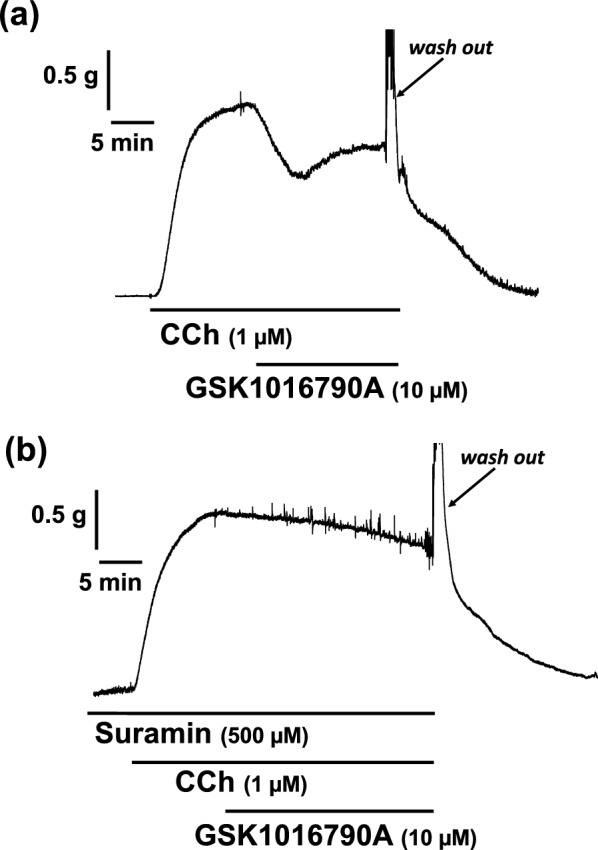
A transient receptor potential vanilloid (TRPV) 4 channel opener-induced relaxation in the rat esophagus. Representative tracings demonstrating the relaxation effect of GSK1016790A (10 µM) on longitudinal tension of the rat esophagus in the absence (**a**) or presence (**b**) of suramin (500 µM), a non-selective antagonist for P2 receptors, are shown. *CCh* carbachol

To investigate whether ATP is released via transient receptor potential vanilloid (TRPV) 4 channel activation endogenously, we examined the effect of a TRPV4 opener on carbachol-induced contractile response. The rat esophageal segment was precontracted with carbachol (1 µM), and then GSK1016790A (10 µM), a TRPV4 channel opener, was applied in the bath. Application of GSK1016790A produced a relaxation (Fig. 7a). After washing out, the tension returned to the basal level. In addition, pretreatment with suramin (500 µM) blocked GSK1016790A (10 µM)-induced relaxations in rat esophageal segments (Fig. 7b).

## Discussion

In the present study, we investigated the characteristics of the mechanical response induced by ATP in the rat esophagus to clarify purinergic regulation of esophageal motility. Our major findings are (1) exogenous application of ATP evoked relaxation of the esophageal smooth muscle, (2) pretreatment with a non-selective P2 receptor antagonist or a selective P2Y antagonist inhibited the ATP-induced relaxation, (3) pretreatment with a blocker of K_ATP_ channels inhibited the ATP-induced relaxation and application of an opener of K_ATP_ channels produced relaxation, and (4) administration of a TRPV4 activator mimicked ATP-induced relaxation. These findings suggest that ATP, which might be released from epithelial keratinocytes endogenously, is involved in inhibitory regulation of the longitudinal smooth muscle in the muscularis mucosae of the rat esophagus via activation of P2Y receptors and then opening of K_ATP_ channels.

The muscularis propria of the rat esophagus is entirely composed of striated muscles [2, 3, 14]. On the other hand, the rat esophagus also contains a smooth muscle layer in the muscularis mucosae [6, 29, 30]. The esophageal smooth muscles in the muscularis mucosae are longitudinally arranged and thus can express longitudinal mechanical responses exclusively [6, 30, 31]. Our results showed that application of ATP evoked relaxation longitudinally under the condition of carbachol-induced contraction of the esophageal segments. The carbachol-induced contraction might be a smooth muscle response because it was inhibited by application of muscarinic receptor antagonists. Hence, it is reasonable that ATP-induced relaxation in the rat esophagus is a smooth muscle activity in the muscularis mucosae.

To determine whether ATP acts on esophageal smooth muscle through neurons, we used tetrodotoxin that can inhibit neuronal activity via blockade of voltage-dependent sodium channels without affecting motor activity of esophageal smooth muscle [6, 8, 14, 31]. Pretreatment with tetrodotoxin did not affect the ATP-induced relaxation. The findings suggest a low probability of commitment of neurons to ATP-induced relaxation.

Pretreatment with application of a non-selective P2 receptor antagonist, suramin [32–34], or a P2Y receptor antagonist, CBF3GA [34, 35], inhibited the ATP-induced relaxation in the rat esophagus, whereas pretreatment with a P2X receptor antagonist, PPADS [36–38], did not inhibit it. Here, we should notice the selectivity of these antagonists for P2 receptors. CBF3GA (synonym reactive blue 2) have been reported to have the selective effects on P2Y receptors [34, 35]. On the other hand, PPADS have been reported to block not only P2X receptors but also P2Y receptors [39, 40]. However, subfamilies of P2Y receptor antagonized by PPADS is limited [39, 40]. Therefore, these pharmacological investigations indicate that P2Y receptors are involved in ATP-induced relaxation of the rat esophagus.

P2Y receptors are GPCRs [16, 17, 21, 22]. Some GPCRs on smooth muscle are linked to potassium channels and induce inhibitory responses [20, 41–45]. We therefore examined the effects of blockers of potassium channels on the relaxation induced by ATP. Pretreatment of voltage-dependent potassium channel blockers and a calcium-dependent potassium channel blocker failed to attenuate ATP-induced relaxation in the esophageal smooth muscle. However, a K_ATP_ channel blocker blocked ATP-induced relaxation. Therefore, ATP might affect esophageal smooth muscle activity via K_ATP_ channel opening.

Opening and closing of K_ATP_ channels are regulated via activation of Gs protein and Gq/_11_ protein, respectively [44]. P2Y1, 2, 4, and 6 are coupled preferentially with Gq/_11_ protein [17]. On the other hand, P2Y11 receptors are coupled preferentially with Gs protein [17]. In line with this, P2Y11 receptors might be associated with ATP-induced relaxation of the rat esophageal smooth muscle via activation of the Gs protein-adenylate cyclase-cAMP-protein kinase A pathway and opening of K_ATP_ channels, resulting in hyperpolarization of smooth muscle [44]. However, there are also some reports that rodents such as rats and mice do not have the receptors [46], which is inconsistent with our findings. On the other hand, several functions of P2Y11 receptors in rats have also been reported [47–54]. Further investigation is required to identify the subtype of P2Y receptors that is involved in the inhibitory regulation by ATP in esophageal motility.

On the basis of the findings presented here, we consider that P2Y receptors and K_ATP_ channels are localized on smooth muscle cells of muscularis mucosae and are involved in the regulation of esophageal motility. In accordance with this, we have shown localization of K_ATP_ channels on smooth muscle cells of muscularis mucosae of rat esophagi [55, 56]. However, it should be noted that P2Y receptors might be located on interstitial cells of Cajal (ICCs). This is because Otsuka et al. reported that ATP might stimulate the ICCs via P2 receptors and then induce relaxation of smooth muscle of the lower esophageal sphincter [57].

In this study, we did not identify endogenous sources of ATP in the rat esophagus. We consider that one of candidate sources of ATP might be epithelial keratinocytes. Mihara et al. reported that ATP is released from epithelial keratinocytes in the mouse esophagus in response to TRPV4 activation [28]. Being consistent with this, administration of a TRPV4 activator mimicked ATP-induced relaxation. In addition, we should also consider the possibility that neurons and glial cells in the myenteric plexus of the esophagus might release ATP.

In addition to the purinergic system, it is notable that we found regulatory roles of K_ATP_ channels in the esophageal smooth muscle in this study. In accordance with this, nicorandil, an agonist of K_ATP_ channels, causes relaxation of smooth muscle in the lower esophageal sphincter of the rat [58]. We previously demonstrated that K_ATP_ channels also contribute to motor regulation of striated muscle in the rat esophagus [55, 56]. We think that elucidation of the coordination between striated muscle and smooth muscle is an important issue in studies on esophageal motor regulation. To address this issue, K_ATP_ channels might play a key role.

There were some variations in response patterns among tissue preparations isolated from the different animals (see Figs. 1b, d, 2a, c and e, 3, 4a, c, 5a, and c). In addition, there were variations in the pharmacological effects of used antagonists. We speculate that these variations might be dependent on unexpected conditions of isolated preparations and animals. The unexpected conditions might be derived from not only artificial technical variations but also physiological conditions in animals. In future, it is necessary to investigate whether this possibility is important for the role of the purinergic regulation in the esophageal motility.

**Fig. 8:**
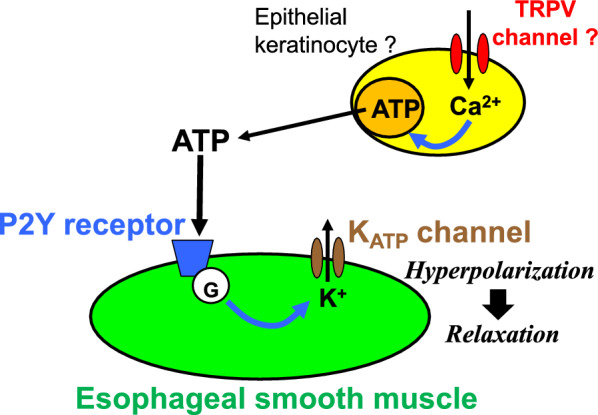
A schema representing purinergic regulation of longitudinal smooth muscle in the muscularis mucosa of the rat esophagus via P2Y receptors and K_ATP_ channels

Generally, it is important to control contraction and/or relaxation in the longitudinal direction separately from those in the circular direction for effective peristalsis of the gastrointestinal tract [18, 59]. Longitudinal motor response plays an important role in the esophageal peristaltic activity and assists effective propulsion [60, 61]. Although the physiological roles of longitudinal smooth muscle in the muscularis mucosa are controversial, some reports indicate that the muscularis mucosa may assist in generating propulsive esophageal motility [29, 62]. So, our findings suggest that the purinergic system may contribute to effective propulsion in the esophagus (Fig. 8).

## Conclusion

The present study clarified that ATP, which might be released from epithelial cells endogenously, induces relaxation responses of longitudinal smooth muscle in the muscularis mucosa of the rat esophagus via P2Y receptors and K_ATP_ channels (Fig. 8).

## Data Availability

The datasets used and/or analysed during the current study are available from the corresponding author on reasonable request.

## Abbreviations

ADP: Aadenosine diphosphate
ATP: Adenosine triphosphate
CBF3GA: Cibacron blue F3GA
GPCRs: G protein-coupled receptors
ICCs: Interstitial cells of Cajal
K_ATP_ channel: ATP-dependent potassium channel
PPADS: Pyridoxal phosphate-6-azophenyl-2,4-disulfonic acid
RT-PCR: Reverse transcription-polymerase chain reaction
SE: Standard error of the mean
TEA: Tetraethylammonium chloride
TRPV: Transient receptor potential vanilloid
4-AP: 4-Aminopyridine
4-DAMP: 4-Diphenylacetoxy-N-methyl-piperidine methiodide
